# Biochemically Constrained Multi‐Omics Integration Reveals Protein–Metabolite Dependencies Across Diseases

**DOI:** 10.1002/advs.77067

**Published:** 2026-08-12

**Authors:** Minghui Zhao, Na Zhou, Ruotong Liu, Xiaofei Li, Jian Li, Fuzhong Xue, Qingzhen Hou

**Affiliations:** ^1^ Department of Biostatistics School of Public Health Cheeloo College of Medicine Shandong University Jinan China; ^2^ National Institute of Health Data Science of China Shandong University Jinan China; ^3^ Department of Otolaryngology‐Head and Neck Surgery Shandong Provincial ENT Hospital Shandong University Jinan China; ^4^ Department of Endocrinology and Metabolism Shandong Second Provincial General Hospital Jinan China

**Keywords:** interpretable neural networks, metabolomics, multi‐omics integration, protein–metabolite dependencies, proteomics

## Abstract

Integrating proteomic and metabolomic data is essential for understanding complex diseases, yet current approaches that rely primarily on statistical associations often overlook the structured biochemical relationships between molecular entities and suffer from discriminative instability in small clinical cohorts. Here, we present ProMetNet, a biochemically constrained framework that incorporates pathway‐derived connectivity from the Reactome database into neural network architecture. By encoding protein–metabolite relationships based on reaction topology, ProMetNet models structured cross‐omics dependencies rather than relying solely on statistical correlations, reducing spurious associations while preserving global molecular context and improving robustness in data‐limited settings. Across four heterogeneous disease cohorts, including Alzheimer's disease, type 2 diabetes, COVID‐19, and glioblastoma, ProMetNet consistently outperforms evaluated multi‐omics integration methods, including MOGONET, P‐NET, PEARL, and MOINER, maintaining high discriminative performance under substantial data downsampling. In addition to classification accuracy, the framework prioritizes biologically plausible protein–metabolite dependencies that are not captured by conventional differential or correlation‐based analyses. Importantly, pathway‐level signals identified by ProMetNet demonstrate consistent discriminative performance in independent large‐scale population data from the UK Biobank (N = 47,507), supporting their robustness and generalizability. Together, these results establish ProMetNet as a biologically grounded and interpretable framework for multi‐omics integration, enabling robust identification of structured molecular dependencies across diseases.

## Graphical abstract

ProMetNet introduces a biologically constrained deep learning framework for proteo‐metabolomic integration by embedding Reactome‐derived pathway topology into neural networks. It captures non‐linear molecular dependencies and pathway‐level metabolic reorganization, enabling interpretable discrimination. The model achieves robust performance under data sparsity and identifies clinically relevant pathway signatures validated across large‐scale population proteomics datasets.

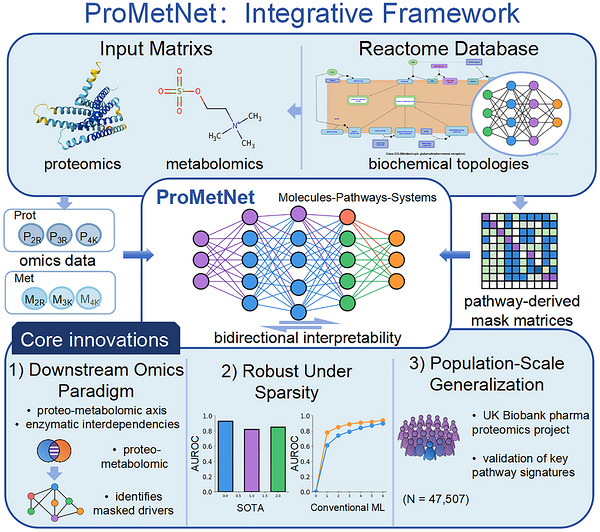

## 1 Introduction

Deciphering the inherent complexity of diseases necessitates a holistic exploration across multiple molecular strata, underscoring the critical need for integrating multi‐omics data to gain comprehensive biological insights. As the primary executors and functional end‐points of biological systems, proteins and their cognate metabolites jointly illuminate the regulatory landscape of disease [1]. For example, the enzymatic interconversion of pyruvate and lactate by lactate dehydrogenase (LDH) is pivotal for NAD^+^ regeneration, which sustains the high glycolytic flux characteristic of the Warburg effect in malignancy [2]. By analyzing proteomics and metabolomics within shared biological pathways, it becomes possible to capture the dynamic interplay between the regulatory (protein) and functional (metabolite) layers. Such integrative analyses can uncover perturbed pathways masked in single‐omics studies, thereby facilitating precise disease subtyping, cross‐omics biomarker discovery, and improved understanding of complex pathologies.

Current multi‐omics integration paradigms are broadly categorized into four strategies [3]: correlation‐based integration (e.g., relying on statistical correlations [4, 5, 6], merging P‐values [7]), dataset concatenation (fusing datasets into a unified matrix [8]), multivariate modeling (employing factor analysis or ensemble modeling across omics [9, 10, 11]), and pathway‐based integration (mapping data to biological pathways to provide functional context). While the former three methods provide statistical utility, they are frequently affected by high‐dimensional noise and limited interpretability, as they predominantly operate on quantitative matrices while overlooking intrinsic biological organization. This detachment from biochemical context often leads to model overfitting and necessitates secondary enrichment analyses to infer biological relevance. In contrast, pathway‐based methods align data with functional knowledge, offering more direct biological interpretability. However, existing methods like ActivePathways [12], DPM [7], and PathIngrate [13] typically treat pathways as static collections of entities. Such simplifications overlook the hierarchical and reaction‐level organization governing molecular systems, thereby limiting a systems‐level understanding of cross‐omics relationships. Furthermore, although advanced frameworks like MOGONET [11] and P‐NET [14] have shown promise, they are primarily designed for upstream omics layers (e.g., DNA methylation and copy‐number variation) rather than the functional protein–metabolite axis, where direct biochemical dependencies play a central role. Consequently, achieving biologically meaningful integration of these downstream layers within an interpretable framework remains a significant challenge.

To address these challenges, we present ProMetNet, an interpretable multi‐omics integration framework based on a pathway‐derived neural network. Unlike conventional deep learning models, ProMetNet utilizes mask matrices constructed from the Reactome database to constrain network connections according to known biochemical relationships. This architecture organizes the data into a functional hierarchy, mapping proteins and metabolites to specific pathways that ultimately connect to high‐level biological processes. By modeling these structured dependencies across hierarchical levels, ProMetNet enhances discriminative performance and interpretability while preserving biologically meaningful organization. Furthermore, we integrated a topologically adjusted SHAP (SHapley Additive exPlanations) [15] algorithm to quantify the importance of features and hidden nodes at each network layer. By ranking contributions of molecular features and pathways, the framework highlights biologically plausible signals and their relationships across levels, enabling a systems‐level interpretation of model outputs.

We evaluated the performance of ProMetNet across four distinct pathologies encompassing varied biofluids: Alzheimer's disease (AD), type 2 diabetes (T2D), COVID‐19, and glioblastoma (GBM). Benchmarking against current multi‐omics integration frameworks (MOGONET, P‐NET, PEARL [16], and MOINER [17]) and conventional machine learning models, ProMetNet demonstrated superior discriminative performance and robustness across varying sample sizes. Beyond this, the framework prioritizes biologically plausible features, including molecules that are not identified by conventional differential analysis, highlighting the sensitivity of the pathway‐constrained approach. Furthermore, ProMetNet identifies disease‐associated protein–metabolite relationships consistent with known biological processes. The generalizability of these prioritized signals is further supported using independent large‐scale proteomic data from the UK Biobank (UK Biobank Pharma Proteomics Project, UKB‐PPP), where they maintain robust discriminative performance across diverse clinical cohorts.

Here, we establish ProMetNet as an interpretable, biologically grounded framework for integrating proteomics and metabolomics. By incorporating biochemical structure into model design, our approach enables the identification of biologically grounded molecular dependencies, supporting biomarker discovery and precision medicine.

## 2 Results

### 2.1 ProMetNet Framework

**FIGURE 1:**
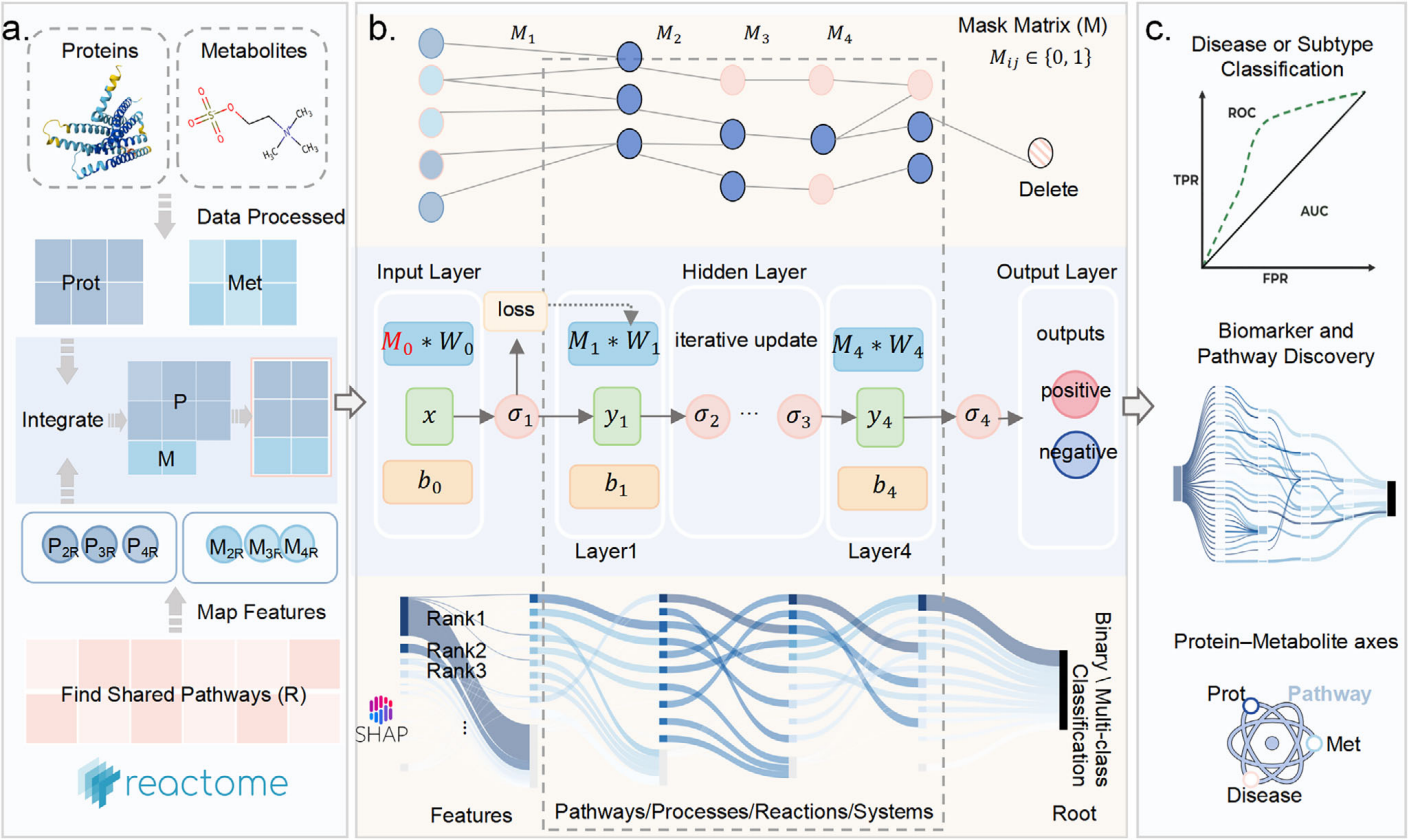
Overview of the ProMetNet. (a) Quantitative protein (P) and metabolite (M) data are systematically integrated based on shared reaction topology from the Reactome database to create a unified, biologically informed molecular profile. (b) This panel details the classification and interpretation workflow. First, Pathway‐derived mask matrices are generated to encode the hierarchical reaction architecture (top). These matrices are then used to construct a sparse neural network, which is trained for accurate disease classification (e.g., AD, T2D, COVID‐19) and subtype identification (e.g., GBM) (middle). Finally, the trained model is analyzed using SHAP to assign functional attribution scores to specific features and pathway nodes (bottom). (c) The framework outputs, enabling high‐performance classification, biomarker discovery, hierarchical pathway analysis, and the resolution of disease‐specific protein–metabolite axes.

The ProMetNet framework provides an end‐to‐end solution for the integration, classification, and biologically interpretable analysis of proteo‐metabolomic data. This process is implemented as a three‐stage computational workflow designed to bridge these two functional omics layers (Figure 1).

The process begins with (i) an omics integration module, which processes quantitative protein and metabolite abundance matrices as primary inputs. Unlike traditional integration methods that independently filter features based on statistical significance, ProMetNet preserves the global molecular context by systematically aligning proteins with metabolites. This module generates two critical outputs: a concatenated molecular feature vector for each sample, and a set of pathway‐derived mask matrices based on prior knowledge from the Reactome database. These matrices translate biochemical pathway architecture into structural constraints, explicitly defining the relationships between molecular entities and their respective biological modules (Figure 1a). By encoding prior biochemical knowledge, the module ensures that the model operates within documented biological contexts and reduces spurious associations that often arise from relying solely on statistical correlations.

These outputs are then passed to (ii) a classification module. In this stage, the pathway‐derived mask matrices are used to construct a sparse, structurally isomorphic architecture. This configuration ensures that all internal connections mirror the biological hierarchy by progressively mapping molecules to biochemical reactions, sub‐pathways, and systemic processes (Figure 1b). By translating biochemical knowledge into this sparse connectivity, the module significantly reduces the number of free parameters and provides a form of structural regularization. This approach improves discriminative stability in clinical cohorts characterized by limited sample sizes, enabling the network to perform high‐accuracy classification for tasks such as disease diagnosis and clinical subtyping. Furthermore, by prioritizing conserved pathway‐level signals over specific molecular identities, this architecture allows for robust generalization even when individual molecular features vary across heterogeneous datasets.

Finally, the trained model serves as the foundation for (iii) a hierarchical interpretability module. This module employs the SHAP algorithm to deconstruct the model outputs by calculating functional attribution scores for each molecular feature and hierarchical pathway node (Figure 1c). This approach enables a top‐down deconstruction of the diagnostic signal, tracing from the systemic disease phenotype back through major pathways and nested sub‐pathways to specific biochemical reactions, and ultimately to the constituent proteins and metabolites. Additionally, because the network layers are directly anchored to the reaction topology, the framework enables quantification of coordinated protein–metabolite signals within shared biochemical contexts, prioritizing biologically plausible pairings and facilitating the interpretation of structured cross‐omics relationships.

Collectively, this workflow establishes ProMetNet as a multi‐omics integration framework that delivers strong discriminative performance while providing biologically grounded interpretability across hierarchical molecular levels, linking specific proteins and metabolites, through perturbed pathways, to disease classification.

### 2.2 Task Design

To evaluate the performance of ProMetNet, we curated four distinct datasets spanning diverse disease etiologies, biofluid sources, and classification objectives (Table 1). The evaluation tasks were designed to cover a broad clinical spectrum: (1) early‐stage discrimination, distinguishing patients with Alzheimer's disease (AD) from those with mild cognitive impairment (MCI) based on cerebrospinal fluid (CSF) profiles; (2) disease diagnosis, differentiating patients with Type 2 diabetes (T2D) or COVID‐19 from controls using plasma and urine, respectively; and (3) molecular subtyping, classifying glioblastoma (GBM) tumor specimens into three distinct molecular subtypes (Proneural, Classical, and Mesenchymal). The baseline characteristics, biofluid sources, and feature distributions for each cohort are detailed in Table 1.

**TABLE 1:** Summary of datasets used for model evaluation.

Disease	Task	Biofluid	Number of features for training					Number of samples for training		
			PRO	MET	CPM	PMN	PRO	MET	CPM	PMN
AD	AD; MCI	Cerebrospinal fluid	6,175	336	1,773	4,563	563	527	511	511
T2D	T2D; HC	Plasma	1,079	228	209	1,082	356	358	339	339
COVID‐19	COVID; Non‐COVID	Urine	2148	433	127	635	90	106	88	88
COVID‐19 Test		Plasma	786	377	339	635	90	75	70	70
GBM	Proneural; Classical; Mesenchymal	Tumor specimen	4,424	63	299	2,958	99	75	75	75

Abbreviations are as follows: AD, Alzheimer's Disease; MCI, Mild Cognitive Impairment; T2D, Type 2 Diabetes; HC, Healthy Control; and GBM, Glioblastoma. The Non‐COVID group includes both healthy individuals and patients with similar non‐COVID symptoms; the “Test” suffix denotes an external validation set. Data types are denoted as: PRO, proteomics; MET, metabolomics; PMN, data integrated by the proposed framework; and CPM, data integrated by correlation analysis.

Notably, despite this clinical heterogeneity, ProMetNet achieved consistently high molecule‐to‐pathway mapping rates (AD: 89.79%, T2D: 94.92%, COVID‐19: 93.54%, and GBM: 96.65%; Figure S4g).

### 2.3 ProMetNet Classification Performance Evaluation

#### 2.3.1 ProMetNet Outperforms Single‐Omics Machine Learning Methods

To quantify the value of multi‐omics integration, we first benchmarked ProMetNet against models trained on single proteomics or metabolomics data. This comparison included the ProMetNet architecture without integration (ni‐ProMetNet) and seven conventional machine learning algorithms: Support Vector Machine (SVM), Random Forest (RF), Naive Bayes, Multilayer Perceptron (MLP), CatBoost, LightGBM, and XGBoost. Model performance was evaluated using k‐fold cross‐validation (*k* = 10, except GBM *k* = 3), utilizing the area under the receiver operating characteristic curve (AUROC) and the precision‐recall curve (PRAUC) as primary evaluation metrics, supplemented by F1‐score and accuracy (Table 2). Comprehensive performance metrics for all models and cohorts are provided in Tables S1–S4.

**TABLE 2:** Comparison of ProMetNet with averaged single‐omics model performance across four diseases.

Disease	Model	ROC‐AUC	PR‐AUC	F1
AD	pm	**0.978** (0.911, 0.995)	0.951	0.905
	p	0.808	0.670	0.448
	m	0.678	0.467	0.228
T2D	pm	**0.994** (0.985, 0.999)	0.995	0.970
	p	0.892	0.897	0.824
	m	0.912	0.924	0.826
COVID‐19	pm	**0.995** (0.986, 0.999)	0.996	0.989
	p	0.890	0.909	0.830
	m	0.945	0.964	0.897
GBM	pm	**0.985** (0.956, 0.998)	0.972	0.940
	p	0.932	0.885	0.823
	m	0.637	0.480	0.455

Across all four disease cohorts, the integrated ProMetNet model consistently achieved higher performance than all single‐omics approaches. In the challenging task of distinguishing Alzheimer's disease (AD) from mild cognitive impairment (MCI), single‐omics baselines yielded modest averages for proteomics (0.808) and metabolomics (0.678), whereas ProMetNet achieved an AUROC of 0.978 (95% CI: 0.911–0.995). In the multi‐class classification of glioblastoma (GBM) subtypes, ProMetNet achieved an AUROC of 0.985 (95% CI: 0.956–0.998), exceeding both the proteomics‐only average (0.932) and metabolomics‐only average (0.637). Higher performance scores for the ProMetNet were also observed in the T2D (AUROC = 0.994, 95% CI: 0.985–0.999) and COVID‐19 (AUROC = 0.995, 95% CI: 0.986–0.999) cohorts, exceeding the corresponding single‐omics averages (Table 2). Even in the COVID‐19 cohort, where metabolomics alone performed strongly (AUROC = 0.945), the integrated model further improved performance.

Together, these results demonstrate consistent performance gains associated with multi‐omics integration. The most pronounced improvements were observed in diseases where single‐omics data, particularly metabolomics, exhibited limited discriminative power, suggesting that the model captures complementary information across omics layers. These findings indicate that ProMetNet leverages cross‐omics signals to achieve robust performance across diverse biological contexts.

Values represent the mean performance metrics from k‐fold cross‐validation with 95% confidence intervals in brackets. pm, ProMetNet model using integrated data; p, model using proteomics data only; m, model using metabolomics data only. The highest value in each ROC‐AUC index is highlighted in bold. Detailed performance metrics, including full confidence intervals, are provided in Table S5.

#### 2.3.2 ProMetNet Outperforms Baseline Integration and State‐of‐the‐Art Methods

To rigorously benchmark the classification capabilities of ProMetNet, we compared its performance against three distinct classes of baseline models: (1) alternative integration strategies, represented by a correlation‐based feature binding network (Corr‐FBN) which inputs correlation‐integrated data into a standard sparse neural network; (2) state‐of‐the‐art multi‐omics integration methods, specifically MOGONET, P‐NET, PEARL, and MOINER; and (3) the comprehensive suite of seven conventional machine learning algorithms utilized in the single‐omics evaluation. All models were evaluated using *k*‐fold cross‐validation (*k* = 10, except GBM *k* = 3).

**FIGURE 2:**
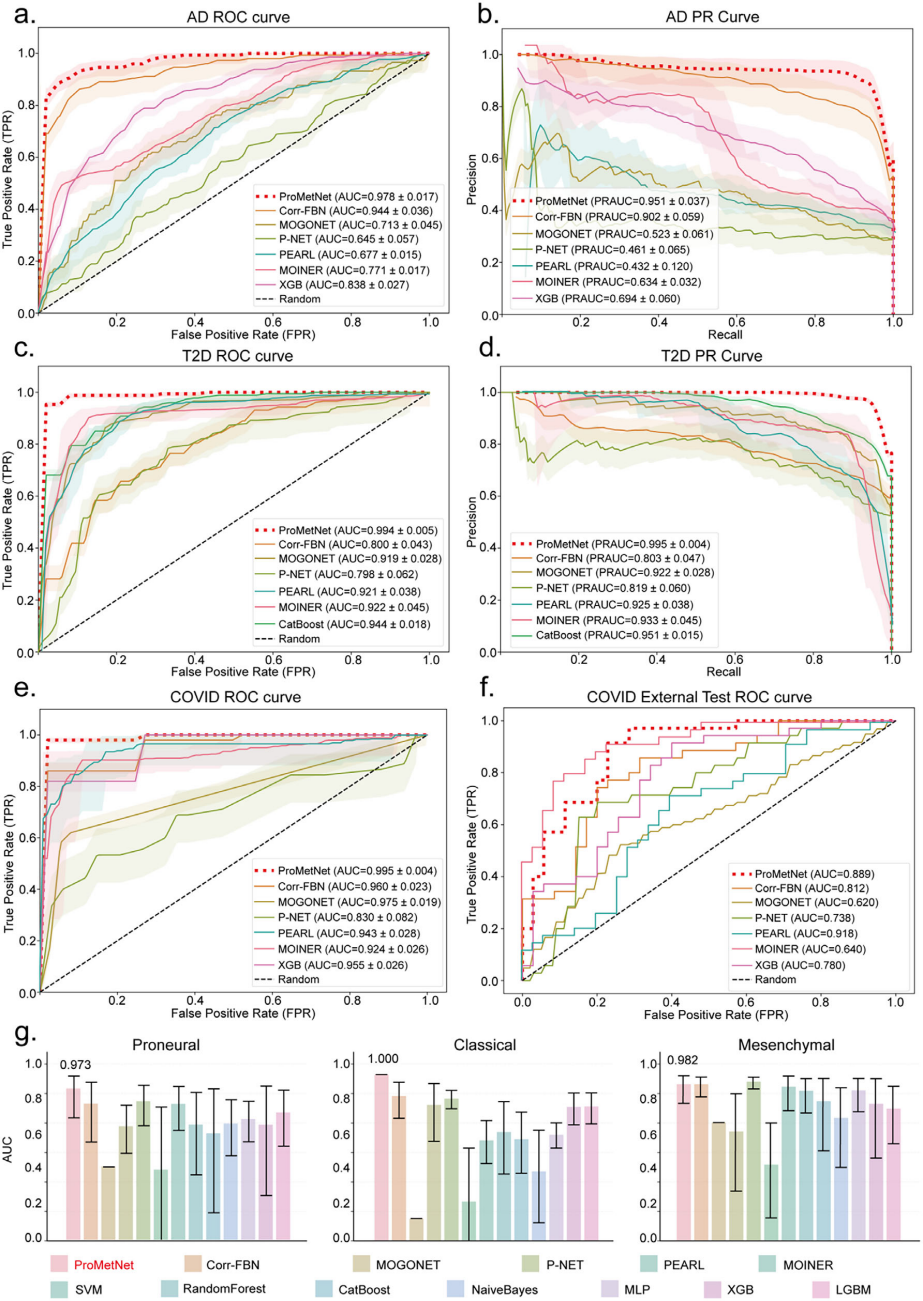
Performance comparison of ProMetNet with baseline integration, state‐of‐the‐art multi‐omics methods, and machine‐learning baselines across different tasks. (a, b) Receiver operating characteristic (ROC) and precision‐recall (PR) curves for the AD task. (c, d) ROC and PR curves for the T2D task. (e, f) ROC curves for the COVID‐19 cohort, displaying internal cross‐validation performance (e) and external test set generalization (f). In these plots (a–f), ProMetNet is shown as a red dotted line; solid lines represent the mean performance from 10‐fold cross‐validation, with shaded areas indicating the standard deviation. (g) Bar plots presenting the mean AUROC for the multi‐class classification of three GBM molecular subtypes (Proneural, Classical, Mesenchymal); error bars represent standard deviation. ProMetNet consistently achieves the highest performance across all evaluated tasks.

Across all evaluated tasks, ProMetNet consistently achieved the highest performance, whereas baseline methods exhibited task‐dependent variability (Figure 2).

In the AD cohort, ProMetNet achieved a mean AUROC of 0.978, outperforming all baseline categories. Notably, while the correlation‐based Corr‐FBN proved to be a strong competitor in this context (AUROC: 0.944), the evaluated multi‐omics integration methods showed lower performance (MOGONET, AUROC 0.713; P‐NET, 0.645; PEARL, AUROC 0.677; MOINER, 0.771; Figure 2a).

Crucially, the T2D cohort highlighted the inconsistent performance of baseline strategies. While MOGONET demonstrated improved adaptability (AUROC: 0.919), PEARL and MOINER also achieved strong performance, with mean AUROC values of 0.921 and 0.922, respectively. These values remained lower than ProMetNet, which maintained a mean AUROC of 0.994. Notably, this performance remained robust even after the exclusion of d‐glucose and d‐mannose (AUROC: 0.987; Figure S1a,b), suggesting that discriminative performance is not driven solely by a small number of dominant metabolites. In contrast, Corr‐FBN, which excelled in the AD task, showed a marked reduction in performance in T2D (AUROC: 0.800), indicating sensitivity of correlation‐based integration to disease context. Across these settings, ProMetNet maintained consistently high performance (Figure 2c).

In the GBM subtype classification task, PEARL also showed strong performance, with a mean class‐wise AUROC of 0.964, whereas MOINER achieved a mean class‐wise AUROC of 0.811. ProMetNet retained the highest mean class‐wise AUROC (0.985), confirming its robust performance in multi‐class molecular subtyping. We further performed an exploratory cross‐biofluid transferability assessment using COVID‐19 cohorts, where models trained on urine‐derived profiles were validated on an independent plasma cohort (Figure S2c). While MOGONET exhibited signs of overfitting with a drop to an AUROC of 0.620 on the test set, ProMetNet retained robust discriminative power (AUROC: 0.889) despite the challenge of cross‐biofluid generalization (Figure 2f). In the internal COVID‐19 cross‐validation task, PEARL and MOINER achieved AUROC values of 0.943 and 0.924, respectively, whereas ProMetNet achieved an AUROC of 0.995. The results for the remaining six machine learning models are provided in Table S6.

In conclusion, ProMetNet's pathway‐based integration is associated with consistent performance advantages over correlation‐based strategies, state‐of‐the‐art multi‐omics methods, and standard classifiers across diverse and complex disease classification tasks.

#### 2.3.3 Robustness to Samples, Preprocessing and Features

To systematically assess the robustness of ProMetNet under conditions of data scarcity prevalent in clinical omics, we evaluated its performance on progressively restrictive subsets of the training data. Using the AD, T2D, and COVID‐19 datasets, we trained ProMetNet alongside seven baseline machine learning models across five distinct data fractions (10%, 20%, 30%, 50%, and 70%). Model stability was rigorously evaluated using a repeated cross‐validation scheme (*k* = 3 for AD/T2D, *k* = 5 for COVID‐19; each repeated three times).

**FIGURE 3:**
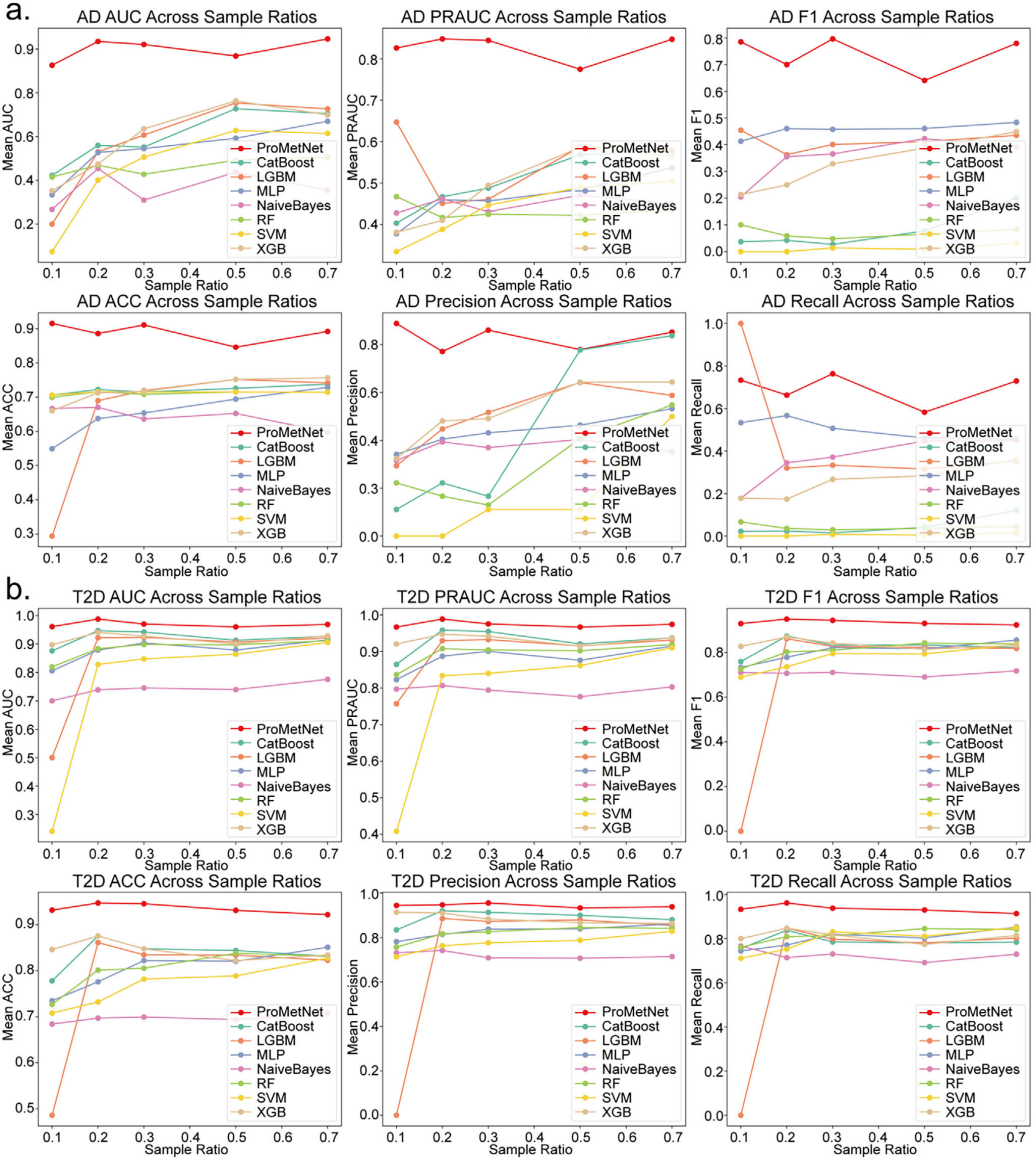
ProMetNet demonstrates robust performance in sample‐limited scenarios. The figure evaluates model performance on the (a) Alzheimer's disease (AD) and (b) Type 2 diabetes (T2D) tasks when trained on progressively smaller subsets of the data (10%, 20%, 30%, 50%, and 70%). Each panel displays six key performance metrics: AUROC, PRAUC, F1‐score, Accuracy, Precision, and Recall. ProMetNet (red lines) is benchmarked against seven baseline machine learning models. The plotted values represent the mean performance from three repeated k‐fold cross‐validations for each sampling proportion. The results illustrate that ProMetNet maintains consistently high classification accuracy even at the lowest sample ratios, exhibiting superior robustness compared to all baseline methods.

The analysis revealed that ProMetNet maintained high discriminative performance even at the most severe downsampling levels, demonstrating greater robustness compared to unconstrained baseline models. This stability was particularly striking in the AD task (Figure 3a), where ProMetNet sustained a robust AUROC of 0.917 using only 20% of the training data. A similar pattern was observed in the T2D cohort (Figure 3b), where ProMetNet maintained a consistently high AUROC (0.961 even at 10% data) across all data fractions, whereas baseline methods exhibited progressive performance decline as sample sizes diminished. Consistent trends were also validated in the COVID‐19 task (Figure S1c). These findings indicate that the pathway‐constrained architecture provides an effective form of structural regularization, supporting stable performance under limited sample conditions.

To further assess robustness to technical variations, we performed three complementary sensitivity analyses using the T2D cohort as a representative dataset. First, we evaluated preprocessing‐order sensitivity by comparing preprocessing performed before data splitting with split‐wise preprocessing, in which imputation and normalization were fitted within the training folds after data splitting. The results were comparable, indicating that preprocessing order had limited impact on model performance. Second, under the split‐wise preprocessing setting, ProMetNet maintained strong performance both without and with log transformation, achieving AUROC values of 0.984 ± 0.013 and 0.963 ± 0.025, respectively, with corresponding PRAUC values of 0.985 ± 0.012 and 0.971 ± 0.018. Third, ProMetNet remained robust across alternative normalization and missing‐data handling strategies, including Z‐score standardization, variance‐stabilizing normalization, median imputation, and MinProb imputation. Detailed results are provided in Table S7 and Figures S2 and S3.

To further examine cross‐biofluid generalizability and the influence of batch correction, we built logistic regression baseline models on unadjusted and ComBat‐adjusted data for urine‐to‐plasma prediction. The unadjusted and ComBat‐adjusted models achieved similar AUROC values of 0.909 and 0.907, respectively, indicating that the cross‐biofluid prediction signal was not primarily driven by ComBat adjustment. Corresponding ROC comparison curves are shown in Figure S3a–c.

Furthermore, we investigated the sensitivity of ProMetNet to feature dimensionality. By ranking features via SHAP values and training models on incrementally larger subsets of top‐ranked molecules, we observed that the model reached near‐optimal performance with a subset of high‐ranked features. Notably, peak classification accuracy was often achievable using a concise subset of high‐importance features rather than the full feature set (Figure S4a–f). This indicates robustness to feature redundancy and supports the effectiveness of SHAP‐based feature prioritization.

### 2.4 Interpretability of ProMetNet

The superior discriminative performance and structural robustness of ProMetNet are supported by an architecture that mirrors the hierarchical organization of biochemical pathways. By incorporating established biological logic into the model design, the framework moves beyond isolated statistical associations to capture structured functional dependencies associated with disease phenotypes. This pathway‐informed structure enables a top‐down deconstruction of diagnostic signals, tracing from systemic manifestations back to specific biochemical reactions. Consequently, the model identifies high‐contribution nodes and non‐linear relationships that remain obscured under traditional statistical thresholds. We performed comprehensive interpretability analyses across all four clinical tasks, with results for COVID‐19 and GBM detailed in the Supplementary Information (Figure S5a,b). In the following sections, we focus on the multi‐level disturbances within the Alzheimer's disease (AD) and Type 2 diabetes (T2D) cohorts, which represent contrasting clinical scenarios of neurodegeneration and metabolic dysregulation.

#### 2.4.1 ProMetNet Identifies Key Pathways Distinguishing AD and MCI

**FIGURE 4:**
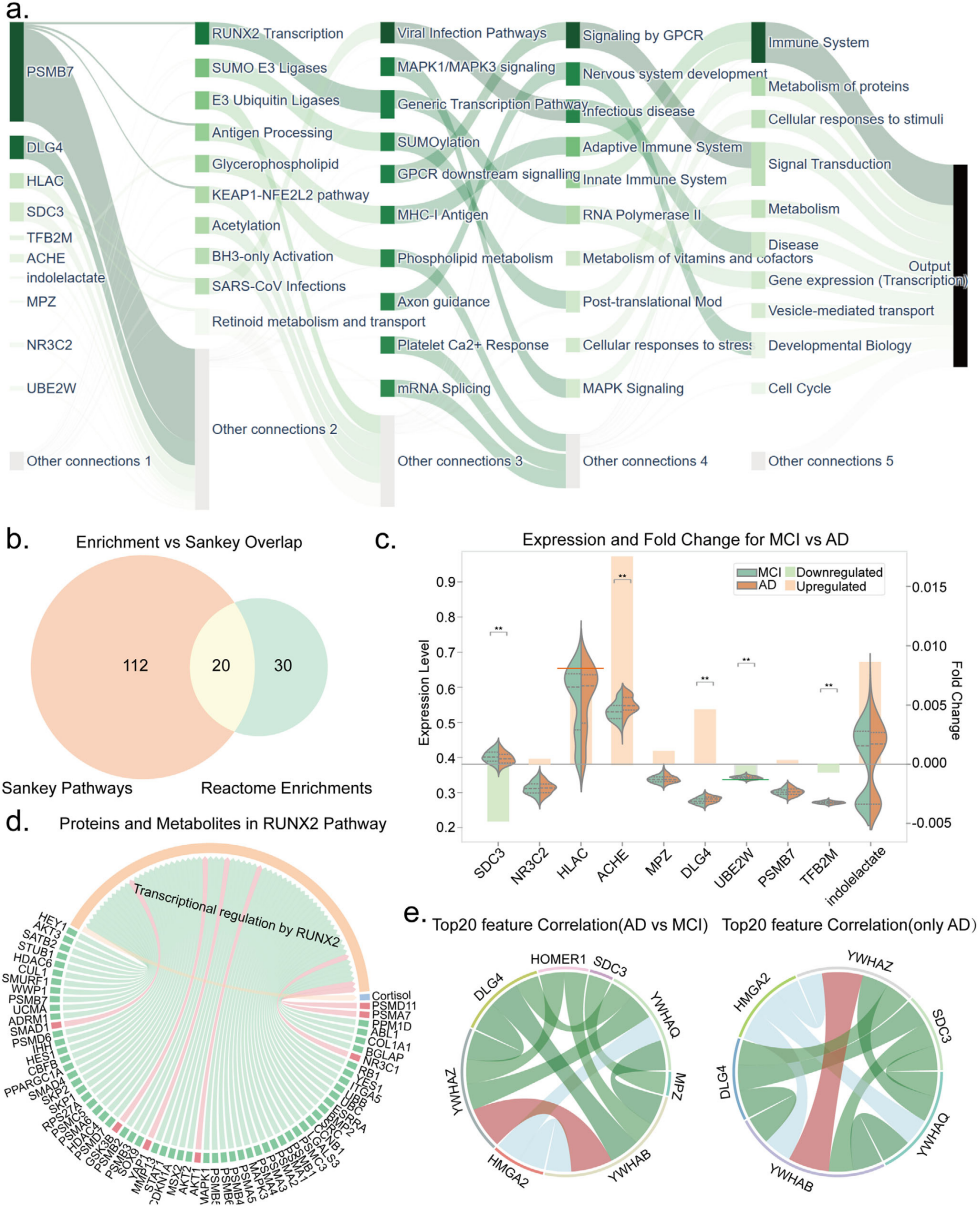
Analysis of Alzheimer's disease associated features and pathways. (a) Multi‐level Sankey diagram illustrating the information flow from input omics features (Layer 1) through intermediate Reactome pathways (Layers 2–4) to final functional systems (Layer 5), where node height and link width are proportional to the SHAP contribution score. (b) Pathway rescue analysis comparing conventional enrichment (FDR < 0.05) versus ProMetNet‐prioritized pathways, highlighting latent signals “rescued” by the model's hierarchical logic. (c) Dual‐axis validation of the top 10 SHAP‐prioritized features, ranked by model importance from left to right. Violin plots (left y‐axis) display expression distributions in MCI (green) and AD (orange) groups, while bar charts (right y‐axis) indicate the magnitude of log2 Fold Change. Asterisks denote statistical significance (adjusted *p* < 0.05); notably, high‐ranking features like PSMB7 lack significance markers, confirming that ProMetNet prioritizes network centrality over simple abundance differences. (d) Chord diagram of the “Transcriptional regulation by RUNX2” pathway, mapping specific proteins (blue), metabolites (orange), and known AD‐associated markers (red) within this regulatory network. (e) Feature independence analysis using chord diagrams of Pearson correlations among the top 20 molecules. Links represent correlations with |*r*| > 0.5 (strong correlations > 0.8 in red), confirming that ProMetNet selects distinct, non‐redundant biological drivers.

To demonstrate the interpretability of ProMetNet, we visualized the model's hierarchical attribution logic in distinguishing AD from MCI using a multi‐level Sankey diagram (Figure 4a). This analysis provides a transparent view of how the model processes biological information, moving from molecular features to systemic functions based on contribution ranking. As shown in the network structure, the information flow originates from the highest‐ranked features (Layer 1), including the proteasome subunit PSMB7, the synaptic marker DLG4, the proteoglycan SDC3, and the metabolite indolelactate. These molecular signals propagate through a multi‐level regulatory network (Layers 2–4), involving key intermediate hubs including RUNX2 Transcription, SUMO E3 Ligases, and Antigen Processing. Finally, these pathways converge into broad functional systems (Layer 5), predominantly the Immune System and Metabolism of proteins, contributing to the final classification output (Figure 4a). This visualization illustrates a coherent mapping from molecular features to higher‐level functional systems.

The distinct advantage of this hierarchical approach is reflected in its ability to resolve signals not detected by conventional statistical thresholds (Figure 4b). Conventional enrichment analysis of the proteomics and metabolomics datasets identified 50 shared pathways (Table S8), none of which reached statistical significance after multiple testing correction (FDR > 0.05). In contrast, by applying a contribution‐based ranking threshold, ProMetNet prioritized 132 functional pathways (SHAP value > 0.05). Notably, 20 of these pathways overlapped with the conventional enrichment results but were retained despite statistical insignificance (FDR > 0.05) by the model's hierarchical logic.

Several of these “rescued” pathways have been associated with AD‐related processes. For example, the “Transcriptional regulation by RUNX2” pathway (FDR > 0.1, normalized SHAP = 0.123, Table S9) was identified as a high‐ranking node (Figure 4d). Figure 4d details the specific protein and metabolite features within this pathway that contributed to the model's decision. Key components in this network include GSK3B [18], a primary kinase implicated in Tau pathology, HDAC6, a regulator of misfolded protein clearance [19], and the stress‐associated metabolite Cortisol [20]. Additionally, the visualization reveals a dense cluster of proteasome subunits (e.g., PSMA and PSMB families).

Similarly, the pathway “SUMO E3 ligases SUMOylate target proteins” (FDR > 0.1, normalized SHAP = 0.101) plays a key role in SUMOylation, a post‐translational modification regulating [21] the stability of proteins such as Tau, BACE1, and HDAC1. Dysregulation of these proteins has been linked to AD‐related processes, including neurofibrillary tangles and Aβ accumulation [22, 23, 24]. Together, these results highlight that ProMetNet prioritizes biologically relevant pathways that may not be detected by conventional statistical analyses.

We further examined the top 10 key molecules ranked by SHAP values (Figure 4c, Table S9). The model successfully identified established markers that showed significant differential expression (adjusted *p* < 0.05), such as ACHE (adjusted *p* = 4.08×10^−10^), SDC3, and DLG4, confirming its alignment with known synaptic deficits in AD [25, 26, 27, 28]. Crucially, however, ProMetNet also assigned high SHAP scores to features that were not statistically significant in univariate analysis (*p* > 0.05), including indolelactate, MPZ, and NR3C2. For instance, PSMB7 received the highest SHAP score despite moderate statistical separation, suggesting its potential importance within the modeled network structure [29]. This suggests that ProMetNet prioritizes features based on their discriminative contribution to the disease network rather than simple abundance differences, thereby uncovering potential biomarkers that are overlooked by standard differential analysis.

Finally, we analyzed Pearson correlations among the top 20 molecules identified by ProMetNet (Figure S4i) to assess their independence (Figure 4e). Most feature pairs exhibited weak correlations (|*r*| < 0.5) in both the combined and AD‐only datasets, with the exception of the YWHA family. This lack of strong linear co‐expression indicates that ProMetNet prioritizes distinct and complementary drivers rather than redundant molecular clusters. Unlike standard correlation‐based methods that select tightly coupled modules, our model captures diverse drivers that represent distinct biological characteristics of AD, ensuring that each feature contributes unique information to the classification task.

#### 2.4.2 ProMetNet Prioritizes Key Biomarkers and Functional Pathways in T2D

**FIGURE 5:**
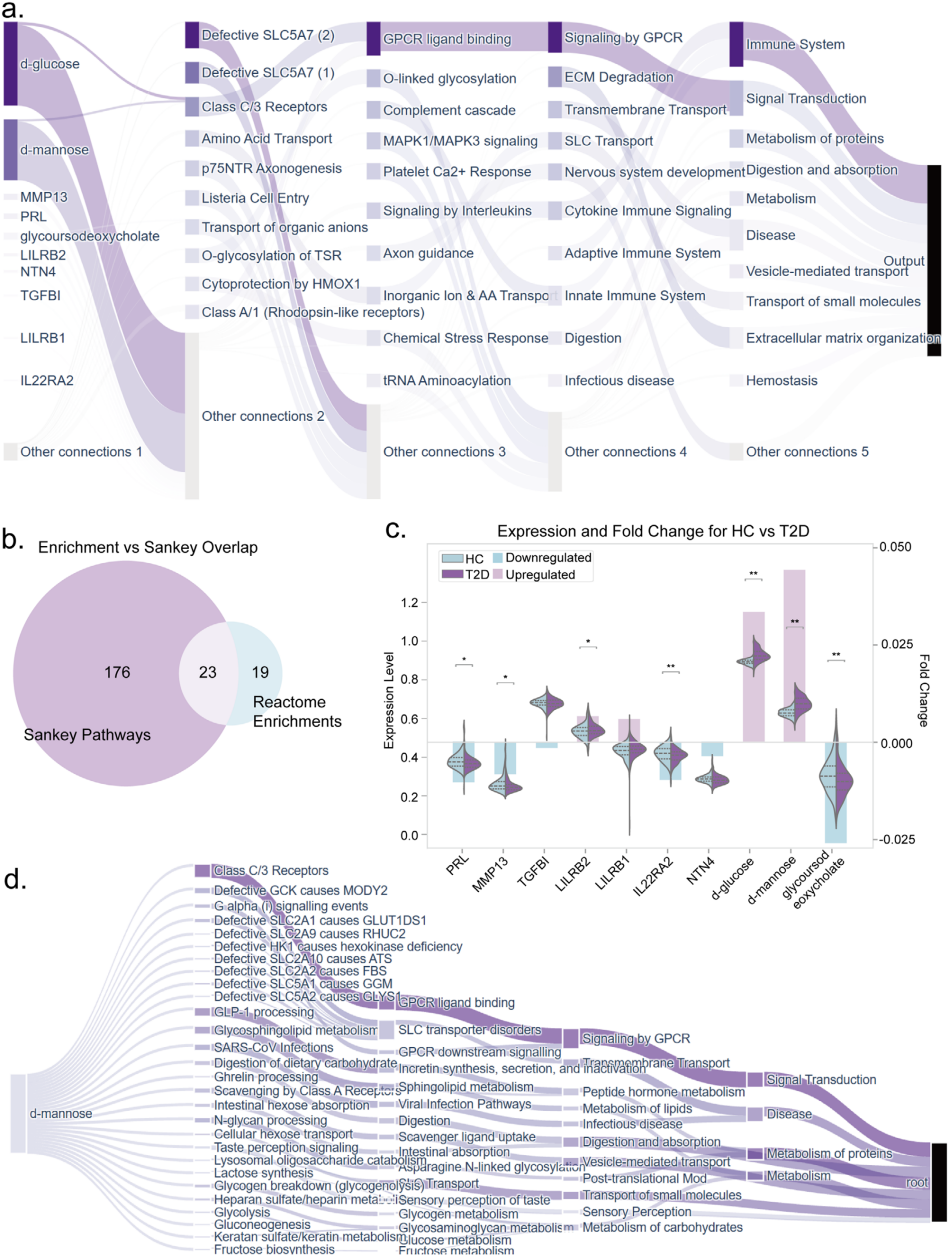
Analysis of Type 2 diabetes (T2D) associated features and pathways. (a) Multi‐level Sankey diagram of the T2D ProMetNet network, tracing the information flow from integrated omics features (Layer 1) through Reactome pathways (Layer 2) to broader functional systems (Layer 3). Node height and link width are proportional to the SHAP contribution score. (b) Overlap analysis comparing conventional enrichment (light blue) versus ProMetNet‐prioritized pathways (purple). (c) Dual‐axis validation of the top 10 SHAP‐prioritized features, ranked by model importance from left to right. Violin plots (left y‐axis) display expression distributions in HC (light blue) and T2D (purple) groups, while bar charts (right y‐axis) indicate the magnitude of log2 Fold Change. Asterisks denote statistical significance (adjusted *p* < 0.05). (d) Case study of feature‐specific interpretability: using d‐mannose as a representative example, this Sankey diagram demonstrates how the model traces an individual biomarker to its specific regulatory pathways.

In this part, we show how our method interprets the prediction results for T2D (Figure 5a). The high‐contribution molecules in Layer 1 include d‐glucose, d‐mannose, glycoursodeoxycholate, MMP13, and LILRB2. Key intermediate hubs in Layers 2–4 are exemplified by “GPCR ligand binding”, “Transport of organic anions”, and “Defective SLC5A7”. Finally, these pathways converge into broad functional systems (Layer 5), predominantly the Immune System and Metabolism, contributing to the classification output.

Specifically, three key metabolites, d‐glucose, d‐mannose, and glycoursodeoxycholate, were identified with high SHAP values, consistent with their known roles in glucose metabolism and energy homeostasis (Figure 5a). All three were also significant in differential expression analysis (adjusted *p* < 0.01), with d‐glucose and d‐mannose significantly upregulated, and glycoursodeoxycholate significantly downregulated in T2D vs. HC (Figure 5c). Guided by the model's global contribution ranking, we zoomed in on the d‐mannose subnetwork (Figure 5d) to resolve its fine‐grained connectivity. The model captured its established involvement in glycolysis and MODY2 (via GCK‐related mechanisms), while additionally connecting it to ‘Class C/3 Receptors’ (GPCR family) and SLC transporter networks. This suggests that mannose‐associated signals may relate to both metabolic imbalance and altered GPCR‐mediated signaling or transmembrane transport in T2D [30, 31, 32]. Similarly, glycoursodeoxycholate downregulation aligned with the ‘Transport of organic anions’ pathway, consistent with previous reports of altered OAT3 (SLC22A8) function in diabetic kidneys [33].

Additionally, ProMetNet also identified several proteins associated with T2D. Among the top 10 molecules identified, PRL and IL22RA2 were significantly different between T2D and HC (adjusted *p* < 0.05) (Figure 5c), consistent with previous validations of PRL in mouse models [34] and IL22RA2 in both mouse and human cohorts [35]. In contrast, LILRB1, TGFBI, and NTN4 did not reach statistical significance in our dataset but are well‐documented T2D‐associated markers in prior literature [36, 37, 38], suggesting that ProMetNet captures latent disease signals that standard differential analysis might miss. The model also prioritized MMP13 (adjusted *p* < 0.05) and LILRB2, proteins implicated in neuro‐inflammatory processes, including MMP13 regulation of BACE1 translation [39, 40] and LILRB2 modulation of Aβ‐TREM2 signaling [41]. Collectively, these results highlight the ability of ProMetNet to identify T2D‐relevant molecules and pathways beyond conventional statistical thresholds.

Separate enrichment analysis of the proteomics and metabolomics datasets identified 42 shared pathways (Table S10); however, statistical significance (FDR < 0.05) was entirely absent in the metabolomics layer and limited to only five pathways in the proteomics layer. In contrast, ProMetNet's ranking logic prioritized 199 functional pathways (SHAP > 0.05). Within the 23 pathways shared between both methods (Figure 5b), the model identified 18 high‐ranking pathways (including “Class A/1 Receptors” and “GPCR ligand binding”) that failed to meet conventional significance thresholds (FDR > 0.05).

These prioritized nodes provide a systems‐level view of metabolic alterations in T2D. The “Defective SLC5A7” pathway (normalized SHAP = 0.151) has been associated with links between choline metabolism and insulin resistance [42]. In parallel, the “Class C/3 Receptors” pathway (normalized SHAP = 0.112) incorporates glucose and mannose as key upstream molecules, both of which are fundamental indicators of impaired glucose homeostasis [43, 44]. Other high‐ranking nodes—including “p75NTR regulates axonogenesis” (normalized SHAP = 0.092), “Transport of organic anions”, and “Cytoprotection by HMOX1” (normalized SHAP = 0.083)—further validate the model through their established roles in T2D metabolic dysfunction [45, 46]. Together, these results indicate that ProMetNet highlights biologically relevant pathways that may not be captured by conventional statistical approaches. The top 10 features ranked by SHAP values in T2D are listed in Table S9.

Finally, we performed a correlation analysis on the top 20 molecules to examine their interrelationships (Figure S4h,j). Overall, proteins and metabolites exhibited weak correlations, suggesting that these features represent distinct data dimensions. However, specific pairs showed significant associations within the T2D group; for instance, PRL and CDH1 displayed a significant negative correlation (*r* < ‐0.3).

#### 2.4.3 ProMetNet Shows Balanced Modality‐Specific SHAP Contributions

To further evaluate whether the integrated representation was disproportionately influenced by the larger number of proteomic features (e.g., 6,175 proteins versus 336 metabolites in the AD cohort), we performed a modality‐specific contribution analysis using SHAP values.

Across the AD, T2D, and GBM cohorts, no significant differences were observed between the distributions of feature‐level SHAP contributions derived from proteomic and metabolomic inputs (Figure S3f–i), despite the substantial disparity in the number of input features. Notably, in the COVID‐19 cohort, metabolomic features exhibited significantly higher SHAP contributions than proteomic features (Figure S3h). This indicates that the metabolomic modality, despite its smaller feature count, can yield disproportionate discriminative power when enriched with disease‐specific biological signatures.

Together, these results demonstrate that feature dimensionality alone does not dictate feature importance within ProMetNet, highlighting the effectiveness of the pathway‐constrained integration architecture across highly imbalanced omics layers.

### 2.5 ProMetNet Reveals Key Protein–Metabolite Axes in Disease Contexts

To translate the model's global hierarchical logic into mechanistic insights, we focused on interpreting the protein–metabolite dependencies prioritized by the model. We utilized high‐contribution metabolites in Layer 1 as “phenotypic anchors” to pinpoint synergistic proteins within refined biological pathways.

#### 2.5.1 Kynurenine Pathway Bias Driven by HAAO in AD Progression

Within the Top 50 features identified by ProMetNet for differentiating AD from MCI (Layer 1), indolelactate (ILA) emerged as the sole metabolite anchor (Table S11). To trace its regulatory origins, we mapped ILA to shared pathways with the top 50 proteins. Crucially, we excluded the overly broad “Metabolism” category to focus on refined modules: “Metabolism of amino acids and derivatives” and “Tryptophan catabolism”.

**FIGURE 6:**
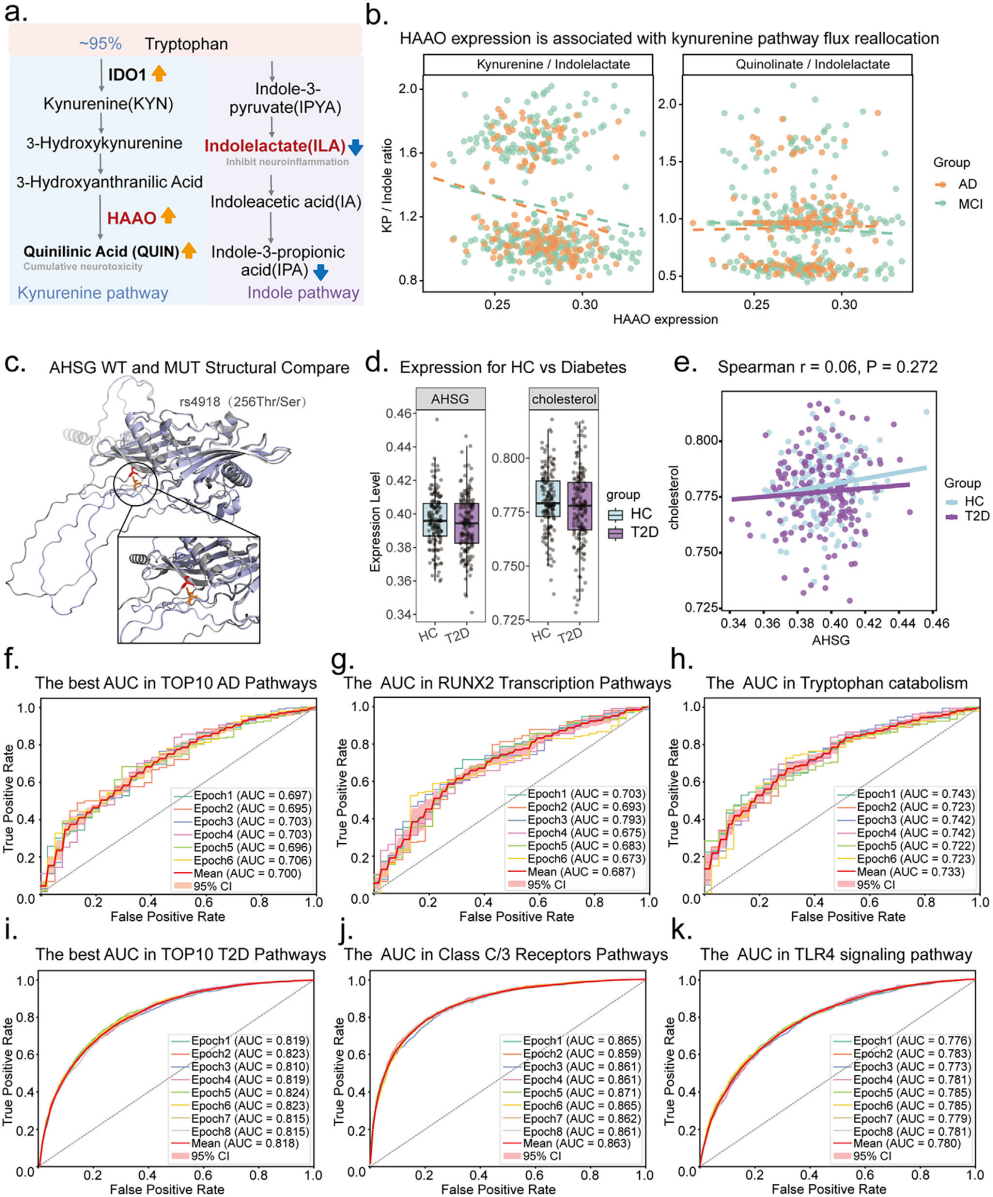
Disease‐specific protein–metabolite axes and pathway validation by ProMetNet. (a) Overview of tryptophan metabolism highlighting the kynurenine (KP) and indole (IP) pathways. Key proteins identified by ProMetNet are marked in red (e.g., HAAO); orange and blue arrows indicate components upregulated or downregulated in AD, respectively. (b) Association between HAAO expression and KP flux reallocation. HAAO expression is plotted against kynurenine/indolelactate (left) and kynurenate/indolelactate (right) ratios in AD (orange) and MCI (green). (c) Structural comparison of wild‐type (WT) and mutant (MUT) AHSG. WT is shown in grey and MUT in light purple; the rs4918‐associated residue is highlighted. (d) Expression of AHSG and cholesterol levels in healthy controls (HC) and T2D. (e) Spearman correlation between AHSG expression and cholesterol in HC (blue) and T2D (purple). (f) UKB‐based pathway validation in AD. Proteins from the top 10 ProMetNet‐identified pathways were mapped to UKB proteomics data and evaluated for AD prediction using logistic regression. (g) Validation of the “Transcriptional regulation by RUNX2” pathway and its downstream targets for AD classification. (h) UKB validation of the Tryptophan catabolism and its downstream pathways for AD prediction. (i) UKB‐based pathway validation in T2D using the top 10 ProMetNet‐identified pathways. (j) UKB validation of the Class C/3 Receptors pathway and its downstream pathways for T2D classification. (k) UKB validation of the Toll‐Like Receptor 4 (TLR4) Cascade for T2D classification.

Analysis of these refined pathways prioritized 3‐hydroxyanthranilate 3,4‐dioxygenase (HAAO, P46952), the critical enzyme responsible for producing the neurotoxin quinolinic acid (QA) [47, 48]. As illustrated in Figure 6a, this axis captures a pattern consistent with a “metabolic shunt”. While IDO1 is generally considered a major regulator of kynurenine pathway flux, ProMetNet prioritized HAAO as a potential downstream node in AD‐related tryptophan metabolism. This computational prioritization is consistent with a testable hypothesis that inflammation‐associated HAAO upregulation may redirect upstream substrates toward quinolinic acid production, thereby contributing to a neurotoxic kynurenine‐biased metabolic state [49, 50, 51]. Crucially, this creates a competitive “tug‐of‐war” for the tryptophan pool: in our AD cohort, HAAO expression exhibited a significant inverse correlation with ILA levels, as well as with the Kynurenine/ILA and Kynurenate/ILA ratios (Figure 6b). This inverse association reflects a flux bias where the neurotoxic kynurenine branch is amplified at the expense of the neuroprotective indole branch. Notably, ProMetNet recovered this specific metabolic state even when conventional pathway enrichment failed to detect global perturbations (Figure S5c,d), highlighting its ability to resolve localized, high‐resolution molecular dependencies.

#### 2.5.2 Generalization of the AHSG‐Centered Lipid–Inflammatory Axis to T2D

To demonstrate the framework's versatility, we evaluated the T2D generalization task. Among the Top 50 features, cholesterol emerged as the most contributory metabolite (SHAP rank 3) after excluding glucose‐related markers. Guided by the “thrombo‐inflammation” paradigm [52, 53], we prioritized molecules bridging inflammatory activation and hemostatic stability. Only fetuin‐A (AHSG, P02765) and SELL were co‐enriched in both “Immune System” and “Hemostasis” pathways, with AHSG exhibiting superior model contribution (normalized SHAP = 0.0049, Table S12).

We integrated structural biology to validate this selection. Circulating AHSG abundance is significantly modulated by the rs4918 (Thr256Ser) missense variant [54], located in the flexible connecting peptide loop (Figure 6c). Structural modeling suggested a modest local conformational change around the rs4918‐associated residue (ΔΔG = −0.34 kcal/mol), providing a computationally supported hypothesis that altered AHSG structure may be related to Fetuin‐A processing or function [55, 56]. Despite the lack of a strong linear correlation between AHSG and cholesterol (Figure 6d,e), ProMetNet prioritized this pair, suggesting a biologically plausible hypothesis consistent with a ‘carrier failure’ model. Physiologically, Fetuin‐A acts as a mineral and lipid chaperone; we hypothesize that genetic or structural alterations of AHSG may affect the stability of cholesterol‐loaded calciprotein particles (CPPs). This interpretation is consistent with previous studies linking CPP dysregulation to TLR4‐mediated inflammation and insulin resistance [57, 58, 59].

### 2.6 Functional Validation of Key Pathways Using UKB Proteomics Data

To evaluate the robustness of ProMetNet‐identified pathways, we performed independent validation using the UK Biobank Pharma Proteomics Project (UKB‐PPP, N = 47,507; 1,463 proteins). For each Sankey‐prioritized top 10 pathway, we assessed the discriminative performance of encompassing protein features within the UKB‐PPP cohort using logistic regression.

In the AD task, the E3 ubiquitin ligases ubiquitinate target proteins pathway achieved an average AUROC of 0.700 (Figure 6f), while the Transcriptional regulation by RUNX2 axis with proteins from this pathway and its downstream pathways reached 0.687 (Figure 6g). For T2D, the Class A/1 (Rhodopsin‐like receptors) pathway, a major therapeutic target class, yielded an average AUROC of 0.818 (Figure 6i). Notably, the Class C/3 Receptors pathway, which was overlooked by conventional enrichment analysis, exhibited superior discriminative power with an average AUROC of 0.868 (Figure 6j).

We further validated whether the identified protein–metabolite axes map to biologically coherent pathways in the UKB‐PPP cohort. For the AD‐associated HAAO–ILA axis, proteins within the tryptophan catabolism and its downstream modules yielded an average AUROC of 0.733 (Figure 6h). For the T2D‐associated AHSG–cholesterol axis, we evaluated the Toll‐like receptor 4 (TLR4) cascade, because lipid‐bound AHSG (Fetuin‐A) has been reported to modulate this inflammatory signaling pathway. Consistent with this functional link, classification based on TLR4 cascade proteins yielded an average AUROC of 0.780 (Figure 6k). Overall, ProMetNet identifies protein–metabolite dependencies that translate into reproducible, pathway‐level discriminative power across large‐scale population data.

## 3 Discussion

Unravelling the molecular complexity of heterogeneous diseases remains a defining challenge in the era of precision medicine. While multi‐omics approaches theoretically offer a holistic view of pathophysiology, bridging the gap between high‐dimensional data integration and biologically interpretable clinical translation has proven difficult. We have developed ProMetNet, an interpretable end‐to‐end framework designed to resolve this bottleneck through a pathway‐informed architecture. By embedding molecular relationships directly into the neural network, ProMetNet achieves bidirectional interpretability: it enables the tracing of specific proteins or metabolites to their downstream functional contexts, while simultaneously allowing for the deconvolution of high‐level biological processes back to their constituent molecules. This architecture not only delivers competitive performance in disease classification and subtype differentiation but also transforms the “black box” of deep learning into a structured framework for generating biologically informed hypotheses for translational research.

Crucially, ProMetNet advances the field by moving beyond simple abundance‐based integration to prioritize functional connectivity. Unlike isolated differential analysis, which often misses subtle systemic shifts, our framework leverages a global feature network to prioritize classification contribution over mean differences, thereby detecting multivariate discriminative signals that are univariately obscured. For instance, in our Alzheimer's disease (AD) cohort, the model prioritized the HAAO‐Indolelactate axis. This feature pair captures a pattern consistent with pathway‐level metabolic reorganization rather than simple co‐expression. Similarly, in the context of Type 2 Diabetes (T2D), the model prioritized the AHSG‐Cholesterol pair despite their negligible linear correlation. This selection highlights the framework's capacity to utilize latent features, demonstrating that the informative weight of a multi‐omics feature often lies in its functional connectivity rather than its statistical magnitude [60, 61].

Our analysis also revealed distinct, disease‐dependent patterns in omics contribution, offering guidance for future multi‐omics study designs. We observed that metabolomics exhibited relatively limited performance compared to proteomics in distinguishing AD from mild cognitive impairment (MCI), a trend consistently reflected within ProMetNet's feature ranking where only a single metabolite, indolelactate, was prioritized among the top 10 features. This signal attenuation likely reflects the blood‐brain barrier (BBB), which may partially decouple systemic metabolic profiles from localized neural pathological changes. However, this does not diminish the intrinsic value of metabolic information. Rather, it suggests that in neurodegenerative contexts, metabolites serve as specific “anchors” for proteomic regulation, whereas in systemic metabolic disorders like T2D, metabolomics drives classification performance comparable to proteomics.

A recurring challenge in multi‐omics integration is the numerical disparity between metabolite and protein features. Previous methods, such as MOGONET, often constrain feature proportions to force artificial balance. In contrast, ProMetNet does not impose rigid thresholds on feature counts. Instead, we adopted a strict pathway‐based inclusion criterion: only proteins and metabolites that co‐occur within the same biological pathway were retained for integration. Specifically, pathways containing only proteins without mapped metabolites, or vice versa, were excluded. To maximize coverage, we integrated pathway annotations from Reactome, supplemented by KEGG. This strategy ensures biological alignment without artificial numerical constraints. Notably, despite substantial clinical heterogeneity, ProMetNet achieved consistently high molecule‐to‐pathway mapping rates (AD: 89.79%, T2D: 94.92%, COVID‐19: 93.54%, and GBM: 96.65%; Figure S4g), confirming the effectiveness of this approach. The shared‐pathway inclusion criterion was designed to support cross‐omics protein–metabolite dependency modeling, rather than to define all disease‐relevant pathways. Protein‐only or metabolite‐only pathways may still reflect meaningful single‐omics disease activity and should be considered complementary to the ProMetNet dependency network. Although ProMetNet showed robustness to common preprocessing choices and missing‐data scenarios, its pathway coverage remains dependent on the completeness and quality of curated biological knowledge bases such as Reactome and KEGG.

Nevertheless, several limitations persist regarding metabolite identification and network construction. A major bottleneck is that metabolites often have disparate identifiers across databases such as HMDB and ChEBI, and these IDs do not always correspond perfectly across systems. For instance, in large‐scale datasets like the UK Biobank (UKB), certain metabolite ratios represent distinct biological patterns yet lack standardized identifiers, precluding their inclusion in pathway‐based models. However, it is important to note that for features where HMDB identifiers were successfully determined, our framework maintained exceptional connectivity within the integrated pathway network, as evidenced by the high mapping rates across diverse cohorts. Future extensions toward multi‐annotation frameworks could effectively address this bottleneck to maximize feature utility.

Furthermore, our current pathway definitions rely on binary (0/1) mask matrices based on the Reactome database to rigorously constrain connectivity. While this binary architecture ensures that the model adheres strictly to documented biological structures, it necessitates the exclusion of pathways or interactions that lack explicit annotation. Recent advances in biomedical data integration have introduced graph‐based learning [61, 62] and multimodal information fusion [63, 64], as exemplified by frameworks such as GREMI [61] and CAMM [64]. These studies provide useful perspectives for incorporating structured molecular relationships and complementary data sources into predictive models. ProMetNet contributes to this methodological landscape by specifically addressing matched proteomic–metabolomic integration and embedding curated reaction‐level biochemical topology as fixed, label‐independent structural constraints. Building on these complementary advances, future extensions may incorporate adaptive graph learning or additional biological information to model previously unannotated protein–metabolite relationships and further expand the applicability of ProMetNet.

In summary, ProMetNet provides a robust and scalable framework for multi‐omics integration. The present study combines multi‐cohort benchmarking, robustness analyses, exploratory cross‐biofluid evaluation, and independent UKB‐PPP pathway‐level validation to support the performance and reproducibility of the framework. Nevertheless, the current validation is subject to the retrospective design and heterogeneity of the available cohorts, biofluids, and assay platforms. To rigorously evaluate model performance and further close the validation loop, future studies should include independent cohorts with matched proteomic and metabolomic measurements, a prespecified and locked analytical pipeline, and standardized head‐to‐head benchmarking using identical preprocessing procedures and data partitions. Orthogonal targeted measurements and functional perturbation experiments will further be required to confirm the biological relevance of the prioritized protein–metabolite axes. These steps will help establish the broader generalizability and translational utility of ProMetNet. Ultimately, by linking disease classification with biologically interpretable insights, our model offers a versatile resource for advancing precision medicine and identifying therapeutic targets supported by structured biological relationships.

## 4 Methods

### 4.1 Data

We evaluated ProMetNet using five analytical datasets representing four disease settings: Alzheimer's disease (AD), type 2 diabetes (T2D), COVID‐19, and glioblastoma (GBM). Following cross‐omics sample alignment, the final integrated datasets comprised 511 samples for AD versus mild cognitive impairment (MCI; AD, *n* = 147; MCI, *n* = 364), 339 samples for T2D versus healthy controls (HC; T2D, *n* = 175; HC, *n* = 164), 88 samples for the COVID‐19 urine development cohort (COVID‐19, *n* = 50; non‐COVID, *n* = 38), 70 samples for the independent COVID‐19 plasma test cohort (COVID‐19, *n* = 35; non‐COVID, *n* = 35), and 75 samples for GBM subtype classification (Proneural, *n* = 28; Classical, *n* = 16; Mesenchymal, *n* = 31). The independent UK Biobank Pharma Proteomics Project (UKB‐PPP) pathway‐level validation included 47,507 participants with 1,463 quantified proteins. Data were sourced from published studies as detailed in Table S13. Cohort‐specific characteristics and sample distributions are summarized in Table 1. Binary classification tasks were implemented for AD (AD vs. mild cognitive impairment [MCI]), T2D (T2D vs. healthy controls [HC]), and COVID‐19 (COVID‐positive vs. symptomatic non‐COVID and healthy controls [non‐COVID]). For GBM, a multi‐class task differentiated Proneural, Classical, and Mesenchymal molecular subtypes as defined by TCGA [65]. Biological hierarchies and pathway‐informed mask matrices were constructed using the Reactome database (accessed April 2, 2025).

### 4.2 Data Processing

To ensure data quality and minimize noise, we applied a preprocessing pipeline to all proteomic and metabolomic datasets. Features with missing values exceeding 20% in any experimental group were excluded. For datasets provided as raw or non‐log‐transformed abundance matrices, log2 transformation was applied when appropriate to stabilize variance; datasets already provided as log‐transformed or normalized matrices were retained in their source‐provided scale. Missing values were then imputed using the K‐nearest neighbors (KNN) algorithm (*K* = 5). To maintain the relative abundance of low‐signal molecules while constraining the influence of outliers, all data were linearly scaled to the [0, 1] range via min‐max normalization. The average expression values were utilized to ensure feature uniqueness. The resulting standardized expression matrices, paired with corresponding clinical labels, served as the primary inputs for model training and evaluation. To assess preprocessing robustness, additional sensitivity analyses were performed in the T2D cohort while keeping all downstream modeling procedures unchanged. These analyses evaluated the influence of preprocessing order, log transformation, alternative normalization strategies (Z‐score standardization and variance‐stabilizing normalization), and alternative imputation approaches under simulated missingness (median and MinProb imputation).

### 4.3 A Pathway‐Informed Interpretable Integration Framework

#### 4.3.1 Integration of Proteins and Metabolites

To construct a biologically grounded feature space, we mapped proteomic and metabolomic data to shared functional pathways. Let P and M denote the complete sets of identified proteins and metabolites, respectively. The quantitative abundance matrices are defined as $E_{p}\in R^{N_{p}\times \left|P\right|}$ and $E_{m}\in R^{N_{m}\times \left|M\right|}$, where N*_p_* and N_m_ represent the sample sizes for each omics layer.

We utilized the Reactome database as the primary topological reference, denoted as the pathway set R. A binary feature–pathway association matrix, $A\in {\left\{0,\;1\right\}}^{\left(\left|P|+|M\right|)\times |R\right|}$, was constructed where A*_i_*_,_ *_j_* = 1 if feature *i* is annotated to pathway *j*, and 0 otherwise. To enforce cross‐omics connectivity, we identified the subset of shared pathways ($R_{\mathrm{shared}}\subseteq R$) that contain at least one protein and one metabolite. Features not participating in any shared pathway were pruned, yielding the refined feature subsets P′⊆P and M′⊆M. The final integrated feature set F is the union of these subsets, with cardinality $\left|F|=\right|P^{\prime }\left|+\right|M^{\prime }|$.

To maximize biological coverage, this integration strategy was applied in parallel using the KEGG database. For KEGG integration, metabolite identifiers were harmonized by mapping KEGG IDs to their corresponding HMDB IDs. The final feature space represents the union of valid features derived from both Reactome and KEGG mappings. This pathway selection step was performed as label‐independent prior construction rather than supervised feature selection. Pathways were retained solely according to Reactome/KEGG annotations and the availability of measured proteins and metabolites, without using disease labels, differential‐abundance results, SHAP scores, or validation/test performance. The resulting pathway‐derived topology was fixed before model training.

Sample alignment was performed by identifying the intersection of subjects present in both omics datasets, denoted as S_common_. The aligned abundance matrices were concatenated to form the unified input matrix $X\in R^{|S_{\mathrm{common}}\left|\times |F\right|}$.

Additionally, to construct a correlation‐based functional biological network (Corr‐FBN), we calculated Pearson correlations between all protein–metabolite pairs. Pairs exhibiting significant high correlation (*r* > 0.5, *p* < 0.05) were selected to generate correlation‐based features.

#### 4.3.2 Construction of the Pathway‐Informed Network

Here, we introduce the ProMetNet framework, which integrates proteomic and metabolomic data to explore disease mechanisms from a multi‐omics perspective. Following feature integration, we structured the hierarchical mapping relationships between molecular features (proteins and metabolites) and lower‐level biological pathways in Reactome, extending to higher‐level biological processes and reactions. Specifically, all mapping relationships were encoded as binary (0/1) mask matrices, providing a topological structure for the network. All pathway‐derived binary mask matrices were constructed before supervised model training and remained fixed throughout all experiments. During model training, only the learnable weight and bias parameters were updated, whereas the binary masks were used solely to constrain the permissible network connections between adjacent layers.

The architecture consists of an input layer, intermediate hidden layers, and a final output layer. The input layer receives the abundance matrices for proteins and metabolites. The hidden layers represent the biological hierarchy, ranging from lower‐level pathways (e.g., regulation of complement cascade) to higher‐level biological processes (e.g., immune system, metabolism) from Reactome, with the number of hidden layers configurable as a hyperparameter based on user‐defined settings. The output layer produces the predicted probability of disease outcomes. Ultimately, the network models the flow of information from fine‐grained molecular features through functional pathways to coarse‐grained biological processes.

The detailed steps for constructing the network are as follows:

(1) Initialization of weight and mask matrices. The weight matrix W is initialized randomly. Mask matrices were constructed according to Reactome mapping relationships through a knowledge‐driven, label‐independent process and were fixed prior to training. This topological design and feature mapping strictly exclude any disease phenotypes. All biological paths are normalized to a fixed n‐layer hidden structure (excluding the output node), where n is specified by the user. To ensure structural uniformity, paths shorter than n layers are extended via node duplication (identity mapping), while paths exceeding *n* layers are pruned from the input side. Paths already satisfying the depth requirement remain unchanged. This standardized *n*‐layer structure generates the mask matrix for each layer, serving as a structural prior, where the initial mask matrix M is derived from the feature‐to‐pathway mapping.

(2) Layer output computation. The output of each layer is calculated as:

(1) $$h^{\left(l\right)}=\;\sigma \left(\left(M^{\left(l\right)}⊙W^{\left(l\right)}\right)h^{\left(l-1\right)}+b^{\left(l\right)}\right)$$

where σ denotes the activation function, M^(^ *^l^* ^)^ is the mask matrix, W^(^ *^l^* ^)^ is the weight matrix, *h* ^(^ *^l‐1^* ^)^ is the input from the previous layer and *h* ^(0)^ = *x* is the input feature matrix, *b* is the bias vector, and ⊙ represents the Hadamard (element‐wise) product.

(3) Backpropagation. Gradients are computed via backpropagation based on the loss function, and network parameters, including the weight matrices are updated using the Adam optimizer to minimize the loss. Binary cross‐entropy is employed as the loss function to quantify the divergence between predicted probabilities and ground truth labels. Concurrently, the fixed, layer‐specific mask matrices, derived from the distinct topological structure of the upstream pathway (1), are applied to the updated weight matrices, thereby enforcing connectivity to the subsequent layer. During backpropagation, the pathway‐derived binary masks were not updated. Instead, they were applied to the trainable weight matrices at each layer so that gradient‐based parameter updates occurred only along curated pathway‐supported connections.

(4) Iterative training. Steps (1)–(3) are repeated iteratively to optimize the weight matrices under fixed pathway‐derived mask constraints until the model converges and achieves optimal performance.

(5) Output layer processing. A sigmoid function maps the final output to a probability value between 0 and 1. Dropout is applied after each layer prior to the activation function to prevent overfitting. The final prediction is obtained by performing a weighted summation across the outputs of all layers, generating the final prediction score $\hat{y}=\frac{\sum_{l=1}^{N}h^{l}}{N}$.

#### 4.3.3 Feature Contribution Calculation and Topological Bias Correction

We employed the SHAP (SHapley Additive exPlanations) algorithm, specifically utilizing the DeepSHAP approximation, to quantify feature contributions. SHAP estimates the Shapley values, which represents the marginal contribution of a feature to the difference between the actual model output and the expected output: S*_n_* = *y* – E(*y*), where *y* = *h* ^(^ *^l^* ^)^ is the attribution value for feature x or the previous layer output *h* ^(^ *^l^* ^−1)^. Here, we use all samples as the background set. The expected model output E(*y*) was computed as $E\;\left(y\right)=\frac{1}{\left|B\right|}\;\sum_{b\in B}\sigma \left(\vec{x_{b}}\right)$ (taking the input layer as an example), where B is the background sample set, $\sigma (\vec{x_{b}})$ is the model' s output probability for background sample. The $\vec{x_{b}}$ is each sample' s feature values. Differences between the model outputs for a specific sample and the expected outputs were backpropagated through each network layer to the input features. Ultimately, each input node received a designated SHAP value S*_n_*.

Features with higher SHAP values do not always reflect true biological importance. Nodes have higher connectivity or centrality often accrue inflated scores, biasing feature evaluation toward structurally prominent nodes rather than biologically significant drivers. SHAP values were corrected to mitigate this topological confounding. Two graph normalization strategies were employed: one based on node degree and the other based on the number of reachable nodes.

Strategy 1: Degree‐based Normalization (DegNorm)

The total degree of a node $d_{tot_{n}}=d_{in_{n}}+d_{out_{n}}$ was defined as the sum of its in‐degree $d_{in_{n}}$ and out‐degree $d_{out_{n}}$. Corrected SHAP values *g*(S*_n_*) were calculated as follows: if the node's degree is higher than *μ*+5*σ* (the average degree of all nodes *μ*) by a certain threshold (e.g., standard deviation *σ*), then the SHAP values were adjusted accordingly $\frac{S_{n}}{d_{tot_{n}}}$; otherwise, the original SHAP values were retained.

(2) $$g\;\left(S_{n}\right)=\left\{\begin{array}{c} \frac{S_{n}}{d_{tot_{n}}},d_{tot_{n}}>\mu +5\sigma \\ S_{n},otherwise \end{array}\right.\;$$

Strategy 2: Reachable Nodes‐based Normalization (SubgNorm)

Define $N_{S_{G_{n}}}$ as the number of nodes n connected to a given node within the entire network structure, i.e., the size of the node's full subgraph. Given that connection complexity grows exponentially in fully connected graphs, the log of $N_{S_{G_{n}}}$ is taken to prevent amplification effects. The corrected node importance *g*(S_*n*_) was denoted. The adjusted node importance is calculated as follows:

(3) $$f\;\left(S_{n}\right)=\frac{S_{n}}{\log\left(N_{S_{G_{n}}}\right)}\;$$

The ProMetNet incorporates two optional SHAP correction methods: DegNorm and SubgNorm. Feature importance was re‐evaluated using these adjusted formulas, enabling the prioritization of top‐ranking molecules (proteins and metabolites) and functional pathways. DegNorm was employed in our analysis. These biologically corrected metrics were unified and are referred to hereafter simply as normalized SHAP values. Hierarchical Sankey diagrams were generated to visualize the relationships between these key molecules and pathways, highlighting feature contributions across different network levels. Additionally, upstream or downstream analyses can be performed based on specific research objectives: downstream analysis traces molecular initiators through biological processes toward disease outcomes, whereas upstream analysis deconvolutes high‐level pathways back to specific molecular drivers.

### 4.4 Training and Evaluation

The ProMetNet was trained using standardized molecular abundance matrices as input and clinical phenotypes as outcomes. Model performance was evaluated via *k*‐fold cross‐validation on the corresponding datasets, with *k* = 10 for the AD, T2D, and COVID‐19 cohorts, and *k* = 3 for the GBM cohort due to its limited sample size. To establish discriminative superiority, ProMetNet was benchmarked against three distinct categories of baseline models: (i) a correlation‐based feature binding network (Corr‐FBN), which integrates multi‐omic data via pairwise correlations before inputting them into a sparse neural network; (ii) representative multi‐omics integration frameworks, specifically MOGONET, P‐NET, PEARL, and MOINER; and (iii) a comprehensive suite of seven conventional machine learning algorithms, including Support Vector Machine (SVM), Random Forest (RF), Naive Bayes, Multilayer Perceptron (MLP), CatBoost, LightGBM, and XGBoost. Classification efficacy was primarily quantified using the area under the receiver operating characteristic curve (AUROC) and the area under the precision‐recall curve (PRAUC), supplemented by F1‐score and accuracy. All methods were evaluated using the same datasets, preprocessed input matrices, data partitions, outcome labels, and evaluation metrics. Hyperparameters were initialized according to the original publications and further optimized within the training data, ensuring that no held‐out fold information was used during model development.

We utilized the COVID‐19 cohorts to conduct an exploratory cross‐biofluid transferability assessment. Specifically, the models were trained on urine‐derived profiles and subsequently assessed using an independent external test set consisting of plasma samples. ComBat adjustment was applied to mitigate dataset‐level distributional differences between the urine and plasma cohorts. Because urine and plasma are physiologically distinct biofluids, this analysis was interpreted as an exploratory cross‐biofluid transferability assessment rather than a conventional same‐biofluid external validation.

Model optimization was performed using the Adam optimizer to minimize the cross‐entropy loss. We incorporated L2 regularization (penalty coefficient λ = 0.001) and applied dropout after each hidden layer to prevent overfitting. To prevent information leakage and overfitting, the entire cycle of model fitting, hyperparameter optimization via grid search (Table S14), and early stopping was executed exclusively within the training data folds. The learning rate was dynamically adjusted based on validation loss derived solely from internal training splits. External test sets and held‐out evaluation folds remained completely untouched during parameter estimation and were reserved strictly for the final objective evaluation of model performance. During model interpretation, SHAP analysis was conducted using the entire dataset as the background reference to quantify the contribution of each node to the model outputs and identify key features driving classification.

### 4.5 Differential and Conventional Enrichment Analysis

We performed differential analysis of proteins and metabolites using the limma R package. Proteins were selected based on a significance threshold of *p* < 0.05, while metabolites were identified using a dual criterion of *p* < 0.05 and Variable Importance in Projection (VIP) > 1. Specifically, we compared the top‐ranked proteins and metabolites identified in the input layer of ProMetNet with those showing significant differential expression. Functional enrichment analysis was performed using the Reactome database Analysis Tools (https://reactome.org/). Proteomic pathways were filtered at FDR < 0.05, and metabolomic pathways at *p* < 0.5 for visualization of enrichment results.

### 4.6 Structural Modeling and Comparison of WT and Mutant AHSG

Human AHSG wild‐type (WT) structure was obtained from the AlphaFold Protein Structure Database (UniProt ID: P02765). The mutant (MUT) AHSG sequence was generated by introducing a T256S substitution at position 256 to represent the rs4918 variant. ColabFold v1.5.5 [66] was employed to predict the MUT structure, incorporating AlphaFold2 with MMseqs2‐based multiple sequence alignment and HHsearch template identification under default parameters. Protein stability changes (ΔΔG) resulting from the rs4918 variant were retrieved from ProtVar. Structural visualization and comparison between WT and MUT AHSG were performed using PyMOL. The WT structure is shown in grey and the MUT structure in light purple. The residue corresponding to the rs4918 variant was highlighted to illustrate local structural differences.

### 4.7 Validation Using UK Biobank Proteomics Data

The UK Biobank Pharma Proteomics Project (UKB‐PPP) dataset comprises 47,507 samples and 1,463 protein features. Binary classification datasets were constructed based on ICD‐10 codes to distinguish AD from MCI, and T2D from controls. Disease classifications utilized specific codes listed in Table S15, including G30 for AD, F06.7 for MCI, and the E11 series for T2D. Individuals with E10, E13, and E14 codes, as well as those with cancer‐related diagnoses, were excluded to form the T2D control group. To ensure a leakage‐controlled evaluation design, each dataset was first partitioned into 70% training and 30% validation sets. Class balancing was then performed exclusively within the training set. For the AD task, balanced 1:1 cohorts were generated through random oversampling of the training set, repeated six times to equalize class sizes. For the T2D task, stratified undersampling was also conducted only within the training set and repeated eight times to ensure comprehensive sample coverage. The validation sets were kept untouched, retained their original clinical distributions, and were used only for final performance evaluation. Performance was evaluated using logistic regression, with age, sex, BMI, and smoking status included as covariates, and model performance was compared using the mean AUROC across validation splits.

## Author Contributions

**Xiaofei Li**: resources, validation. **Na Zhou**: data curation, investigation, writing – original draft. **Ruotong Liu**: investigation, writing – original draft, data curation. **Jian Li**: validation, resources. **Fuzhong Xue**: project administration, resources. **Qingzhen Hou**: conceptualization, methodology, writing – review and editing, supervision, funding acquisition, project administration. **Minghui Zhao**: visualization, writing – original draft, methodology, conceptualization, software, formal analysis.

## Funding

This work was supported by National Natural Science Foundation of China (Grant No. 82473733) and Shandong Provincial Natural Science Foundation (ZR2024ZD18).

## Conflicts of Interest

The authors declare no conflicts of interest.

## Clinical Study Registration

Not applicable. This study involved secondary analyses of previously collected datasets and did not involve prospective participant recruitment or an interventional clinical trial.

## Supporting information




**Supporting File 1**: advs77067‐sup‐0001‐FigureS1‐S5.docx.

**Supporting File 2**: advs77067‐sup‐0002‐TableS1‐S15.xlsx.

## Data Availability

The datasets analyzed in this study are available from their original sources under the applicable access conditions. Alzheimer's disease (AD) data were obtained from the Alzheimer's Disease Neuroimaging Initiative (ADNI) database and are available to qualified investigators upon application through the ADNI data‐sharing platform (https://adni.loni.usc.edu/). COVID‐19, type 2 diabetes (T2D), and glioblastoma (GBM) datasets were obtained from previously published studies (PMIDs: 35026155, 39160153, and 36732634, respectively). Reactome pathway hierarchies were downloaded from the official Reactome repository (https://reactome.org/download‐data) on April 2, 2025. The UK Biobank study was conducted under the generic ethical approval from the NHS National Research Ethics Service (North West Multi‐centre Research Ethics Committee, Ref. 11/NW/0382). All participants provided written informed consent for data collection, storage, and use for research purposes. Individual‐level data from the UK Biobank are available to bona fide researchers upon application to the UK Biobank (https://www.ukbiobank.ac.uk/).
