# Supplementary material for: Biochemically Constrained Multi‐Omics Integration Reveals Protein–Metabolite Dependencies Across Diseases
Source: Adv Sci (Weinh). 2026 Aug 12:e77067. Online ahead of print. doi: 10.1002/advs.77067 (PMC13464550; doi:10.1002/advs.77067)
Supplement: Supplementary file 1 [file ADVS-9999-e77067-s001.docx]

Supporting Information

**Biochemically constrained multi-omics integration reveals protein–metabolite dependencies across diseases**

*Minghui Zhao, Na Zhou, Ruotong Liu, Xiaofei Li, Jian Li, Fuzhong Xue, Qingzhen Hou**

**Figure S1.** Robustness of ProMetNet against critical feature removal and data sparsity.

(**a**, **b**) Receiver operating characteristic (ROC) curves (a) and Precision-Recall (PR) curves (b) for the type 2 diabetes (T2D) classification task following the targeted exclusion of two key signature metabolites. ProMetNet (red dotted line) retains superior performance relative to all baseline multi-omics and machine learning models. (**c**) Model stability under data sparsity in the COVID-19 cohort. Six performance metrics (mean AUC, PRAUC, F1-score, accuracy, precision, recall) are plotted against the training data ratio (10% to 70%). ProMetNet (red line) maintains strong and stable performance across all sampling levels.

**Figure S2.** Robustness evaluation of T2D binary classification under split-sample preprocessing pipelines.

All pipelines follow split-wise processing: the full dataset is first divided into training and test folds, after which normalization and missing-value imputation are performed separately. (**a**) ROC curves and corresponding AUC values from split-wise preprocessing without log₂ transformation. (**b**) Precision–recall (PR) curves and corresponding PRAUC values for the log-free split-wise pipeline shown in panel (a). (**c**) ROC curves and corresponding AUC values from split-wise preprocessing incorporating log₂ transformation. (**d**) PR curves and corresponding PRAUC values for the log-integrated split-wise pipeline shown in panel (c).

**Figure S3.** Evaluation of model robustness against preprocessing variations and modality-specific feature contributions. ComBat sensitivity analysis in urine-to-plasma COVID-19 prediction

(**a, b**) Principal component analysis (PCA) score plots of COVID-19 samples before (a) and after (b) batch effect correction using ComBat. (**c**) ROC curves comparing logistic regression classification performance on cross-fluid COVID prediction. Models trained on unadjusted and ComBat batch-adjusted multi-omics features were evaluated on independent test data to demonstrate the effectiveness of batch correction. (**d**) Comparison of average AUC between ProMetNet and other optimal machine learning models under the T2D dataset across different data scaling methods. The x-axis represents the data scaling methods, and the y-axis indicates the average AUC. (**e**) Impact of random data missingness and imputation strategies on model performance under the T2D dataset. The x-axis indicates the missing data ratio, and different colors represent distinct imputation methods. (**f-i**) Statistical evaluation of the distribution differences in feature importance (measured by absolute SHAP values of non-zero features in the input layer, source layer == 0) between different omics modalities across four datasets: (f) AD, (g) T2D, (h) COVID, and (i) GBM. Statistical significance is annotated within the plots (*** p < 0.001, Mann-Whitney U test).

**Figure S4.** Extended performance evaluation, feature mapping rates, and molecular correlation profiles.

(**a–f**) Discriminative performance of ProMetNet and baseline machine learning models across different feature count thresholds. Metrics include mean AUROC for Alzheimer's disease (AD; a), type 2 diabetes (T2D; c), and COVID-19 (e), and mean F1-score for AD (b), T2D (d), and COVID-19 (f). (**g**) Feature mapping rates across four clinical cohorts. High and consistent mapping rates (AD 89.79%, T2D 94.92%, COVID-19 93.54%, GBM 96.65%) demonstrate that the pathway-constrained inclusion criterion efficiently retains most informative functional features. (**h**) Feature independence analysis based on Pearson correlations among the top 20 prioritized molecules in the T2D cohort. Links denote correlations with |r| > 0.3 (r > 0.8 highlighted in red). (**i**, **j**) Top-ranked molecular features from layer1 top 20 for clinical state discrimination. Panel (i) distinguishes mild cognitive impairment (MCI, green) from AD (orange); panel (j) differentiates healthy controls (HC, light blue) from T2D patients (purple).

**Figure S5.** Sankey diagram and conventional pathway enrichment.

(**a**, **b**) Discriminative information flow from molecular features through nested Reactome pathway layers to final diagnostic output in COVID-19 (a) and glioblastoma (GBM; b) cohorts. Node and edge widths scale with SHAP contribution scores. In COVID-19, the model highlights pathways related to immune regulation, viral infection, and metabolic homeostasis. In GBM, it identifies key drivers including organelle function, autophagy (mitochondrial and lysosomal clearance), and membrane trafficking coupled with endocytosis. (**c**, **d**) Conventional Reactome pathway enrichment analysis of differentially expressed proteins (c) and metabolites (d) in the Alzheimer's disease (AD) cohort. Bar lengths denote statistical significance ($\text{-lo}\text{g}_{\text{10}}\text{(FDR)}$ for proteomics and $\text{-lo}\text{g}_{\text{10}}\text{(P)}$ for metabolomics), and color gradients represent the number of mapped molecular entities per pathway.
